# New Advances in Human Thermophysiology

**DOI:** 10.3390/life12081261

**Published:** 2022-08-18

**Authors:** Jose Ignacio Priego-Quesada

**Affiliations:** 1 Research Group in Sports Biomechanics (GIBD), Department of Physical Education and Sports, University of Valencia, 46010 Valencia, Spain; j.ignacio.priego@uv.es; 2 Biophysics and Medical Physics Group, Department of Physiology, University of Valencia, 46010 Valencia, Spain

Human thermoregulation is the integrative physiological responses of the body used to maintain a core temperature with values close to 37 °C, even in a wide range of activities and environments [1]. The human body aims to maintain a balance between heat production and heat loss [2]. These mechanisms are coordinated by the hypothalamus, receiving thermal input from the skin and core, nonthermal input from the cardiovascular control centre, the brain and muscle mechanoreceptors and metaboreceptors, and will use this information to generate outputs that modify cutaneous circulation or eccrine sweating, among others [3]. However, certain conditions may not always be compensable either due to exercise intensity, individual characteristics or/and extreme environmental conditions. In this sense, although these complicated scenarios are of great interest in recent years, helping us to assess impact and prevention strategies for high-level athletes during events such as the Olympic Games Tokyo [4], the Doha World Athletics Championships [5] or the Soccer World Cup in Qatar [6], human thermophysiology is present in diverse applications (e.g., occupational ergonomics).

The papers published in this Special Issue discuss the topics of greatest concern to the population and the scientific community from the perspective of human thermophysiology. Most of the articles evaluate how the physiological response during or after exercise is affected by extreme environmental conditions, such as high temperatures [7], hot and humid environments [8,9], dehydration status [10] or even cold environments [11]. This issue is of concern to society as a whole, since global warming is an established threat, greatly impacting our environment [12]. An example of this is that the increase in the intensity, duration and frequency of heat waves leads to a high number of deaths, especially in vulnerable populations [13].

The articles published in the Special Issue “Human Thermophysiology” in *Life* have provided interesting results that allow a more specific understanding of how human beings behave during or after physical exercise in extreme environmental conditions. Heat and high humidity can reduce muscle recruitment as a method of preventing body hyperthermia [8]. In this sense, a lower gradient between the core and skin temperature could be responsible for a reduction in the performance [8], in agreement with previous studies [14,15]. On the other hand, Melo-Marins et al. showed how dehydration decreases glutathione levels, which have an important antioxidant property [10]. A novel result of these authors showed that erythrocytes may contribute to the release of glutathione during exposure to heat stress [10]. Chabert et al. also showed how the temperature of the air breathed in while cycling in a hot and high humidity environment could impact physiological and perceptual responses [9]. In this sense, breathing air at 23 °C compared to 33 °C improves performance capacity and reduces discomfort [9]. Last but not least, a pilot study suggested that in cold environmental conditions, the running performance was reduced [11].

All these studies are important for understanding factors that limit performance and developing effective prevention strategies. Baillot et al. recommended methods to decrease skin temperature or mimic cold sensations (e.g., menthol vaporization on the skin during exercise) during exercise in hot and high-humidity environments [8]. In addition, the results of Chabert et al. could recommend the modification of the breathed air temperature during aerobic exercise [9]. On the other hand, adequate warm-up, hydration, training acclimatization, and clothing strategies for cycling under cold ambient conditions are recommended [11]. Concerning prevention strategies, it has recently been investigated whether the evaluation of changes in baseline skin temperature could be a good indicator of muscle damage. Although some results have been promising, many other studies have shown that after a muscle damage protocol, a marathon or a half marathon, there is no change in baseline skin temperature [16,17,18]. In this sense, the study published in this Special Issue by Rojas-Valverde et al. showed an increase in baseline skin temperature the day after a marathon in a hot environmental condition [7]. As a lack of relationship was observed between this increase in skin temperature and muscle damage markers, the authors suggested that this is the result of the physiological process after the marathon (e.g., an increase in endothelial nitric oxide or glycogen resynthesis). Its effect on the skin temperature can be observed because the peripheral vasodilation is promoted by the hot environment [7].

Another current topic that has been investigated in this Special Issue is the expression of browning adipose tissue. The factors that explain the activation of brown adipose tissue, in a situation of exercise or baseline conditions, are of great interest, as it is a possible therapeutic target for people with obesity [19]. The findings of Nintou and colleagues showed that in vitro models are effective for assessing the crosstalk between adipocytes and contracting muscle, and therefore changes in the expression pattern of proteins related to the browning of adipose tissue [20].

Finally, assessing thermal comfort is of great interest for estimating the conditions where most humans would feel comfortable in an everyday environment [21]. Mekjavic and colleagues presented a strong correlation between the perception of thermal comfort and the behavioural regulation of thermal comfort, which was not proved by previous studies [21]. Moreover, they showed that gender and age are two important factors that explain thermal comfort variability [21]. These results are important to consider in future thermal comfort evaluations.

The purpose of this Special Issue was to highlight relevant works and the latest updates on human thermophysiology. Science must continue to provide knowledge regarding the physiological and thermoregulatory bases of the human body, as well as measurement methods and new applications in which thermoregulatory analysis is of interest. Great advancements have been achieved in understanding the physiological response during exercise in extreme environments, the assessment of thermal comfort, and the evaluation of the expression of browning adipose tissue in vitro.
